# An exploratory study on transformational leadership competencies in global health

**DOI:** 10.3389/frhs.2026.1876328

**Published:** 2026-08-04

**Authors:** Barbara M. Löfflad-Bürkin, Katarzyna Czabanowska, Marta Vicente-Crespo, Suzanne Babich, Núria Casamitjana, Luis Eugenio De Souza, Laetitia Rispel, Elizeus Rutebemberwa, Anja Matthiä, Fredros Okumu, Nino Kuenzli, Axel Hoffmann, Marco Waser, Rajesh Kamath, John P. Ehrenberg, Jan Hattendorf, Julia Bohlius

**Affiliations:** 1 Education Department, Swiss TPH, Allschwil, Switzerland; 2 University of Basel, Basel, Switzerland; 3 Department of International Health, Institute of Care and Public Health Research, Maastricht University, wHO CC for Public Health Leadership and Workforce Development, Maastricht, Netherlands; 4 Department of Health Policy Management, Institute of Public Health, Medical College, Jagiellonian University, Krakow, Poland; 5 Research and Related Capacity Strengthening Division, African Population and Health Research Center, Nairobi, Kenya; 6 School of Public Health, Faculty of Health Sciences, University of the Witwatersrand, Johannesburg, South Africa; 7 Department of Community and Global Health, Richard M. Fairbanks School of Public Health, Indiana University Indianapolis, Indianapolis, IN, United States; 8 Education and Training, ISGlobal, Barcelona, Spain; 9 Department de Medicina, Facultat de Medicina I Ciències de la Salut, Universitat de Barcelona (UB), Barcelona, Spain; 10 Collective Health Institute, Federal University of Bahia, Salvador, Bahia, Brazil; 11 South African Research Chairs Initiative, School of Public Health, Faculty of Health Sciences, University of the Witwatersrand, Johannesburg, South Africa; 12 African Field Epidemiology Network (AFENET), Kampala, Uganda; 13 Department of Health Policy, Planning and Management, School of Public Health, Makerere University, Kampala, Uganda; 14 School of Biodiversity, One Health and Veterinary Medicine, University of Glasgow, Glasgow, United Kingdom; 15 Department of Environmental Health and Ecological Sciences, Ifakara Health Institute, Ifakara, Tanzania; 16 Swiss School of Public Health (SSPH+), Zurich, Switzerland; 17 Directorate, Swiss TPH, Allschwil, Switzerland; 18 Department of Healthcare and Hospital Management, Prasanna School of Public Health, Manipal Academy of Higher Education, Manipal, India; 19 Arboretum Frutales Mayas Non-Governmental Organization Cholul, Mérdia, Mexico

**Keywords:** transformational leadership, workforce development, competency-based education, doctoral education, equity

## Abstract

**Background:**

PhD programs typically emphasize scientific competencies but may insufficiently prepare graduates to lead complex global health challenges. Persistent inequities, weak governance and emerging threats to global peace demand leaders who can critically assess systems, respond to local needs, and foster equitable, sustainable change. Such leadership requires reflexivity, humility, and collaborative learning. This study explores how PhD graduates across world regions perceive transformational leadership competencies and compares competencies developed during PhD training with those relevant in their current workplace.

**Methodology/findings:**

Using an expert-validated survey based on a previously published competency framework and Delphi-derived behavioral descriptors (CROSS-guided; internationally piloted), we surveyed 618 graduates from Swiss TPH, CARTA, SSPH+, and CAPHRI; 71 graduates from 27 countries responded (response rate 11.5%). Median age was 45 years (IQR 41–51), 49% were women; median time since graduation was 6 years (IQR 3–9.5). One third completed their PhD in Africa and two thirds in Europe. Most worked in education (53.5%) or health care (10%), with others in NGOs, government, or international organizations. Competencies were rated highly relevant in the workplace (means 5.58–6.23, 1–7 scale). However, mean ratings (5.29–5.97) suggest that PhD training contributed less to competency development than the level relevant in the current workplace. Graduates from low- and middle-income countries reported better alignment between training and workplace relevance than those from high-income countries. Remaining in one location during the PhD was associated with higher perceived development. There was no evidence for gender effects.

**Conclusions/significance:**

Transformational leadership competencies are highly valued in the workplace but not consistently developed through PhD training, particularly in high-income countries. Programs with formally structured leadership-related educational components and dedicated pedagogical approaches were associated with higher levels of self-reported competency development. These findings should be interpreted cautiously given the low response rate and reliance on self-reported data. Future research is needed to determine curricular elements that contribute to leadership competency development. A transformational leadership lens may provide a useful framework for strengthening doctoral education in global health while incorporating lessons from low-income settings.

## Introduction

Persistent challenges such as unequal power structures, weak governance, and emerging threats to global peace and the international order require leaders who can critically assess systems, respond to local needs, and drive equitable and sustainable change (1, 2). This need is especially evident in global health, which can be conceptualized as a problem space focused on achieving equity, both within and across countries (3). In this context, transformational leadership competencies, as defined in a recently developed competency framework for transformational leadership in public health (4), are increasingly recognized as essential for addressing contemporary global health challenges (5–7).

Many PhD programs may not adequately instill the behaviors relevant for leadership roles that demand proactive, context-sensitive, and locally relevant action. As a result, doctoral training has potentially not kept pace with the challenges of our time and does not sufficiently promote mutual learning and an understanding of global interdependence (8–10). Structural asymmetries within the global health ecosystem, including inequalities between institutions in low- and middle-income and high-income countries, can shape whose expertise is valued and who has opportunities to lead (11, 12). Sustainable and equitable outcomes benefit from leadership approaches that emphasize critical thinking, informed decision-making and emotional intelligence (1, 13–15). To better prepare future leaders, doctoral education may need to incorporate more practice-oriented and context-specific approaches to strengthen participants' ability to question entrenched structures, build transformative agency, redistribute decision-making power, and drive inclusive change (16, 17).

Previous literature highlights that although local contexts influence how competencies are enacted, core professional capabilities such as navigating complex health systems and addressing global health challenges remain universally relevant and essential across diverse health systems and professional contexts (9, 15). Yet little is known about how well PhD training develops these competencies and whether graduates perceive them as relevant in their professional roles. To address this, we surveyed graduates of health sciences-related PhD programs from low-, medium, and high-income countries. We aimed to (i) assess the perceived relevance of transformational leadership competencies in professional practice and (ii) explore differences between competencies graduates reported developing during their PhD training and those they require in their current work across organizational settings, work areas, and country income groups.

## Methods

### Study design

We conducted a survey that adhered to the CROSS (Consensus-based Checklist for Reporting of Survey Studies) guidelines (18). The four stages of questionnaire development were as follows. (i) We based our initial questionnaire on a published competency framework and its behavioral descriptors, which were developed and validated through a previous Delphi study involving expert consensus (4) (see Supplementary Box S2, S3). For our initial questionnaire, we iteratively refined the structure, wording, and content of these published competence descriptors [see Supplementary Box S3]. (ii) Research team members selected ten PhD candidates from our international partner programs to pilot the survey questionnaire. Our sample was heterogeneous and similar to the demographic of our target population. These ten candidates gave us feedback on survey length, clarity, and structure. (iii) We invited 16 experts who had co-developed the Competency Framework for Transformational Leadership in the original Delphi study (4) to consolidate the transformational leadership section of our survey questionnaire. To accomplish this, experts worked in pairs to synthesize behavioral descriptors and determined the most essential items and narrowed the list (on file). (iv) We invited all study partners to review the draft questionnaire and provide feedback. We asked them to focus on the options we listed for responses, and to affirm that they were clear and relevant.

### Sampling characteristics

To capture a broad global perspective, we contacted four institutional partners that offer health sciences-related PhD programs across a network of 25 universities and five programs (see Supplementary Box S1, Table S2) to reach graduates in the respective programs. The institutional partners included the Swiss Tropical and Public Health Institute (Swiss TPH, Associated Institute of the University of Basel), the Swiss School of Public Health (SSPH+), the Consortium for Advanced Research Training in Africa (CARTA), and the Care and Public Health Research Institute (CAPHRI) at Maastricht University. The included PhD programs vary both in structure and thematic focus, encompassing structurally organized PhD programs as well as open PhD formats. The Inter-University Graduate Campus (IGC), hosted by SSPH+, primarily serves as a hub for high-quality PhD courses, training, and academic exchange in public health. The PhD Program in Public Health (PPH), also hosted by SSPH+, focuses on strengthening doctoral research skills in public health, while the PhD Program in Health Sciences (PPHS), offered by the University of Basel, targets capacity development for PhD participants in the health sciences. CARTA, jointly led by the African Population and Health Research Center (APHRC) in Kenya and the University of the Witwatersrand (Wits) in South Africa, aims to build an African academy for world-class multidisciplinary public and population health research and to develop internationally recognized research leaders (19). CAPHRI, affiliated with Maastricht University, focuses on improving quality of life through innovation in healthcare and public health research. The estimated total graduate pool for Swiss TPH, CARTA, and SSPH+ with valid email addresses consisted of 789 individuals. Data protection regulations limited our access to graduate data across institutional partners. The survey was directly sent via e-mail to Swiss TPH graduates; for all other programs, our partners reached out to graduates via their own channels (e.g., email and LinkedIn). We used purposive, convenience-based sampling to contact graduates via alumni contact lists.

### Data collection methods

The final survey questionnaire had four sections: (i) Participant characteristics; (ii) PhD experience; (iii) Mobility, career, and current working situation and position; and (iv) Rating of transformational leadership competencies. We derived competency ratings from two key questions: “How relevant are the transformational leadership competencies to your work?” and “To what extent has your PhD training as a whole contributed to the development of transformational leadership competencies?”. Sections one, two, and three combined included 26 questions. Section four assessed ten transformational leadership competencies, each represented by distinct behavioral descriptors. For each competency, we asked respondents to rate behavioral descriptors on two items on a Likert scale from one (lowest) to seven (highest). Rankings indicated how much their PhD training had contributed to developing the behaviors and how relevant each behavior was in their current workplace. The questionnaire included open-ended and multiple-choice questions.

We administered the survey online via the EvaSys platform, a web-based, GDPR-compliant survey tool (version: 2025.1, Build: 2654, EvaSys GmbH, Lüneburg, Germany). A student assistant supported technical implementation and setup of the final version within EvaSys [see Online Data]. We expected respondents would take about 20–30 min to complete the questionnaire. To ensure anonymity, we did not collect contact information such as names or addresses. We stored all data securely on the EvaSys (20) platform; access was limited to the research team. Access to the online survey was provided via a single survey link that was distributed through partner program contacts. These contacts included one person from CARTA, one person from SSPH+, and a communications expert from Maastricht. In addition, we reached out directly to Swiss TPH graduates.

### Survey administration

Survey administration was a six-stage process: (i) we held an initial meeting to inform partner contacts about the study and survey procedures, including the reminder schedule, and followed up with detailed email instructions; (ii) we identified contact persons at partner universities and programs; (iii) we provided detailed information about the planned survey via email or telephone, emphasizing the benefits of participation; (iv) we implemented institution-specific recruitment procedures and allowed respondents three weeks to complete the survey. We invited Swiss TPH graduates directly by email and sent two reminders. SSPH+ graduates were invited by email via SSPH+, with one reminder. CARTA program personnel invited graduates, sent two reminders, and promoted the survey through a newsletter announcement. CAPHRI graduates were contacted via LinkedIn through CAPHRI partners. Reminders were sent one week before the deadline for Swiss TPH, SSPH+, and CARTA, and on the final day of the survey period for Swiss TPH and CARTA.

### Ethical considerations

This study involved human participants who completed an anonymous, voluntary online survey about their experiences in PhD training programs. The survey did not collect health-related or other sensitive personal information. The Ethics Committee Northwest and Central Switzerland (EKNZ) determined that this study does not fall within the scope of the Swiss Human Research Act (HRA; Art. 2, para. 1), as it does not constitute research on diseases or the structure and function of the human body. Therefore, ethical approval was not required and the EKNZ was not responsible for the formal review of the project (Req-2020-01425). We exported the de-identified dataset from EvaSys (20) so responses could not be linked to individual respondents. All data was stored securely on the EvaSys (20) platform; only the research team had access.

### Statistical analysis

After exporting survey data from EvaSys (20), we imported them into R (v4.5.1) for exploratory analysis and checked for implausible or inconsistent entries. We summarized the survey population based on proportions, frequencies, or median IQR, as appropriate. Likert-scale items were numeric, and we summarized them as arithmetic means. To compare subgroups, we grouped participants according to their countries of origin' World Bank income group (accessed July 2025; https://datahelpdesk.worldbank.org). We then derived new variables to capture relevant distinctions in the data. For organization type, we grouped respondents as academic if they were employed at an educational institution and as non-academic if they worked in other sectors, including government, health care, NGOs, or international organizations. Because items within each competency showed high internal consistency (Cronbach's alpha 0.77–0.90), we used the grand mean (the pooled mean of the means of the individual items) for most exploratory analysis. We used general linear models to assess differences between the perceived workplace relevance of competencies and the perceived level of competencies developed during PhD training across subgroups, and quantified effect sizes using Cohen's d. Although Likert scale variables represent ordinal categories, we treated them as continuous which is considered appropriate if the scale comprises more than five items (21). We investigated model assumptions and robustness via QQ-plots and Cook's distance. Only 1.9% of the data were missing. No imputation of missing data and no adjustment for multiple comparisons was done.

### Pilot study

Of the ten PhD candidates we invited, five responded (three men and two women). They were Dutch, Colombian, Nigerian, South African, and Zimbabwean and were affiliated with the University of Basel (two candidates), the University of Bern (one candidate), and Maastricht University (two candidates). We found that our first draft of the questionnaire took our candidates 45–60 min to complete, which was longer than we had expected. Respondents recommended we shorten the competency descriptions, remove language that encourages self-reflection, reword the questions to focus on ranking the relevance of competencies, and reduce response time by combining the two rating questions per competency into one. Experts from the original Delphi study condensed and refined behavioral descriptors for each competency and reduced them to the five items they judged most essential. The final version of the competency framework for transformational leadership comprised ten competencies and 46 behavioral descriptors (four or five per competency) (see Supplementary Box S3).

## Results

### Survey dissemination and participant response

At Swiss TPH, we identified 481 email addresses and invited them to participate in the survey. Of these, 104 (21.6%) returned delivery errors (e.g., delivery failure, timed-out email, or undeliverable email); 377 graduates received the invitation. We identified 184 email addresses in the CARTA and 124 in the SSPH+ networks. We assumed an undeliverable rate for these programs comparable to that observed at Swiss TPH (21.6%), corresponding to an estimated 40 undeliverable emails for CARTA and 27 for SSPH+. We estimated that CARTA could reach 144 graduates and SSPH+ 97. We recruited Maastricht graduates via LinkedIn, so we could not quantify this group, but we included these graduates in our overall sample. We estimate that 618 graduates across the three quantified networks received the survey invitation; of these, 71 graduates from 27 countries of origin responded (11.5% response rate) (see Supplementary Figure S1, Box S4).

### Profile of survey respondents

Of the 71 respondents, about half (49%; *n* = 35) identified as women (see Table 1); median age was 45 years (IQR = 41–51); median time since PhD completion was 6 years (IQR = 3–9.5). Based on World Bank income classifications of countries of origin, 22.5% (*n* = 16) came from low-income countries, 43.7% (*n* = 31) from lower-middle-income countries, 7.0% (*n* = 5) from upper-middle-income countries and 26.8% (*n* = 19) from high-income countries. About one third (35.2%; *n* = 25) obtained their PhD at an African university (mainly in Kenya, Nigeria, and South Africa), while most (59.2%; *n* = 42) graduated from European institutions (mainly in Switzerland). CARTA was the most commonly attended PhD program (38.0%; *n* = 27), followed by PPHS (29.6%; *n* = 21). About a third (31.0%; *n* = 22) had been funded by a regular PhD salary, 22.5% (*n* = 16) by a scholarship (e.g., Swiss Government Excellence Scholarships), 21.1% (*n* = 15) by work in project-based positions, and 8.4% (*n* = 6) financed their PhD through personal savings or with the support of family, friend. 21.1% indicated (*n* = 15) mainly other sources.

**Table 1:** Participant characteristics.

Characteristics	N	Percentage
Total	71	100%
Sex		
Women	35	49%
Men	36	51%
Median 45 years		
IQR: 41, 51		
Years since PhD completion		
Median: 6 years		
IQR: 3, 9.5		
**Country Income Group (World Bank classification)** ^a^		
High-income countries	19	26.8%
Lower-middle-income countries	31	43.7%
Upper-middle-income countries	5	7.0%
Low-income countries	16	22.5%
**Geographical Region of PhD education**		
Africa (Kenya, Malawi, Nigeria, Ruanda, South Africa, Uganda)	25	35.2%
Europe (Switzerland, The Netherlands)	42	59.2%
Other	4	5.6%
**PhD program**		
Consortium for Advanced Research Training in Africa (CARTA)	27	38.0%
Care and Public Health Research Institute (CAPHRI), Faculty of Health, Medicine & Life Sciences, Maastricht University	2	2.8%
PhD Program Health Sciences (PPHS), University of Basel, Swiss TPH	21	29.6%
Swiss School of Public Health PhD Program in Public Health (PPH)	8	11.3%
Swiss School of Public Health Inter-University Graduate Campus (IGC) with certificate	5	7.0%
Other	7	9.9%
No structured program	8	11.3%
**PhD funding source**		
PhD salary (taxed income)	22	31%
PhD scholarship (tax-free allowance, e.g., Swiss Government Excellence Scholarships)	16	22.5%
Paid work during PhD time (e.g., employment)	15	21.1%
Own financial savings	3	4.2%
Financial support of family, friends, others	3	4.2%
Mainly other	15	21.1%

a https://datahelpdesk.worldbank.org/knowledgebase/articles/906519 (accessed: 08.07.2025).

### Employment at time of survey

At the time of the survey, most respondents were working in educational institutions (53.5%; *n* = 38). Others held positions in the health care sector (9.9%; *n* = 7), NGOs (9.9%; *n* = 7), governmental organizations (8.5%; *n* = 6), or international organizations (7%; *n* = 5). A few (11.3%; *n* = 8) either worked in commercial enterprises, foundations, or as self-employed, or were unemployed. Of the three work areas they could choose from, most reported working in research and development (64.1%; *n* = 46), public health and epidemiology (53.5%; *n* = 38), or capacity strengthening and training (33.8%; *n* = 24). Additional areas included healthcare (23.9%; *n* = 17), strategy and leadership (21.1%; *n* = 15), and operations and management (15.5%; *n* = 11). Few respondents reported they worked in marketing and communication, policy and administration, quality management, or advocacy (≤4% each). Table 2 and Supplementary Figure S2 summarize organizational settings and working areas. The largest clusters were in Research and Public Health (*n* = 26), Training and Public Health (*n* = 14), and Research and Training (*n* = 16). Smaller clusters appeared between Research and Operations (*n* = 7), while most other combinations showed limited overlap [see Supplementary Figure S3].

**Table 2:** Current working context of graduates.

Working context	N respondents	Percentage of respondents
Employment type	71	100%
Governmental organization [Governmental ministry, governmental department or organization (e.g., education, finance)]	6	8.5%
Educational institution [Universities, educational institutions (e.g., schools, educational facilities)]	38	53.5%
Health care sector [Hospitals, medical practices, health care networks, health insurance, research institutions (without pharmaceutical industry)]	7	9.9%
International organizations (e.g., UN, African Union, WTO, EU, OECD, ASEAN) or bilateral international organization (e.g., USAID))	5	7.0%
National/Local Non-Governmental Organizations (NGOs) or Civil Society Organizations (National or local non-governmental organization (NGO) or civil society organization, non-profit organizations (e.g., charities, social services)	7	9.9%
Other (Not employed, Self-Employment, Foundation, Commercial Organizations)	7	11.2%
Working areas (up to 3 answers possible)		
Strategy and leadership (Performance management, organizational development, strategic decision making, etc.)	15	21.1%
Research and development (Basic/applied research, product development, etc.)	46	64.8%
Operations and management (Program planning, implementation, management/evaluation, procurement, etc.)	11	15.5%
Marketing and Communication (Sales, marketing, communication)	2	2.8%
Organizational management (Human resources, organizational development)	3	4.2%
Policy and administration (Policy planning, public administration)	3	4.2%
Capacity strengthening and training (Education, training, capacity building)	24	33.8%
Quality management and monitoring (Monitoring, evaluation, quality improvement)	3	4.2%
Public Health and epidemiology (Epidemiology, disease control, communicable/non-communicable diseases, health services)	38	53.5%
Healthcare (Primary, secondary and tertiary health care)	17	23.9%
Other	4	5.6%

### Exposure to leadership training during and after PhD

During their PhD studies, 40.8% (*n* = 29) of respondents reported receiving leadership training, which was usually delivered by universities or PhD programs (72.4%; *n* = 21; Supplementary Table S1). Courses or seminars that typically comprised 30 h or less were the most common formats (72.4%; *n* = 21), followed by lectures (37.9%; *n* = 11), and individual coaching (13.8%; *n* = 4). After they completed their PhD, 35.2% (*n* = 25) of respondents reported more leadership training, often through courses or seminars (72.0%; *n* = 18). Some of this post-PhD training was provided by employers (60.0%; *n* = 15); others pursued leadership training independently (48.0%; *n* = 12). Across all formats, post-PhD leadership training generally totaled 30 h or less (see Supplementary Table S1).

### Workplace relevance of transformational leadership competencies

**Figure 1:**
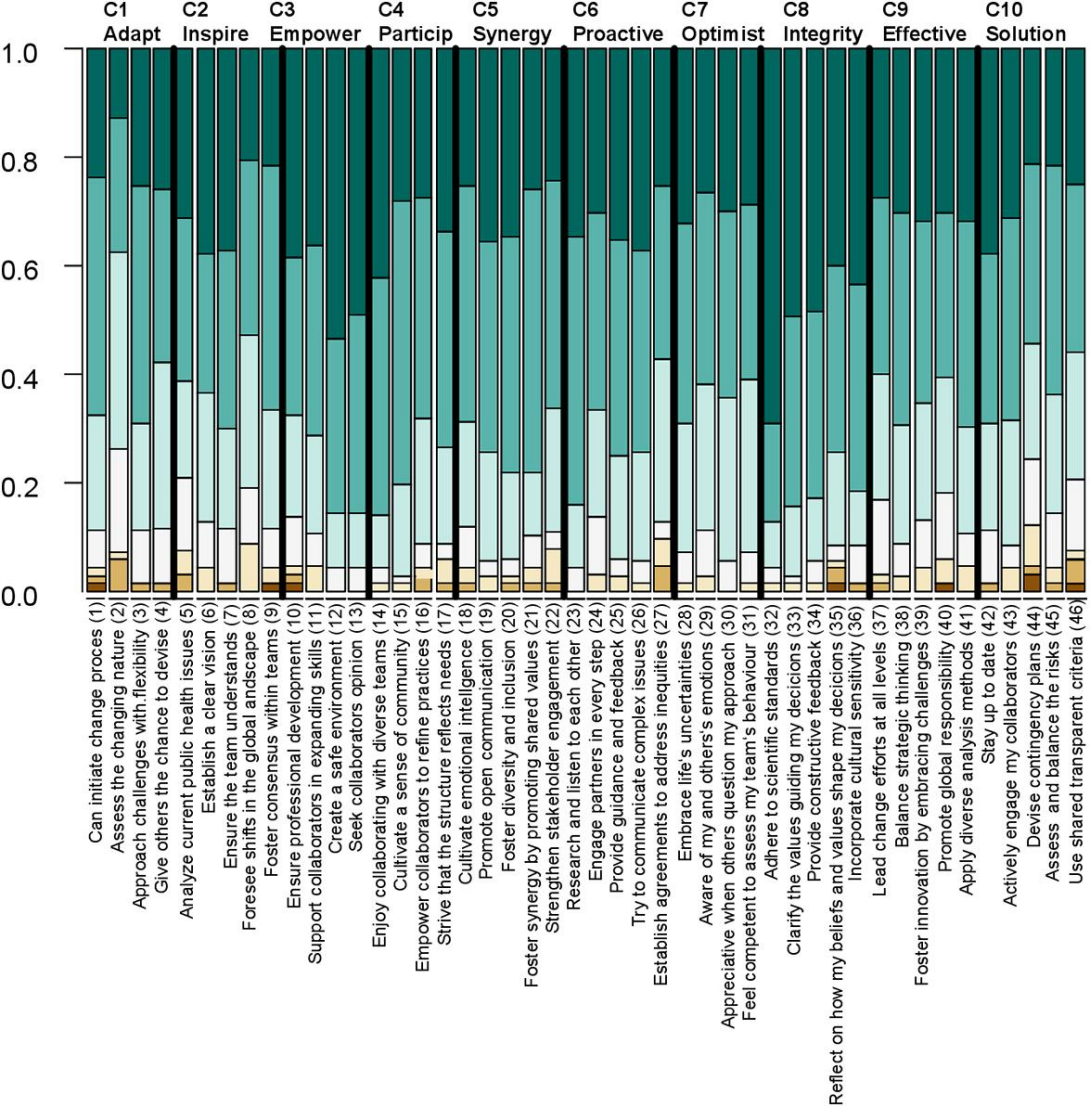
Perceived relevance of competencies in the workplace. Distribution of ratings of perceived relevance of 46 leadership behaviours. Each bar represents one leadership behaviour (see Box S3) rated on a 7-point Likert scale. Bar segments show the proportion of responses at each rating level (yellow = 1 not important, green = 7 very important).

**Figure 2:**
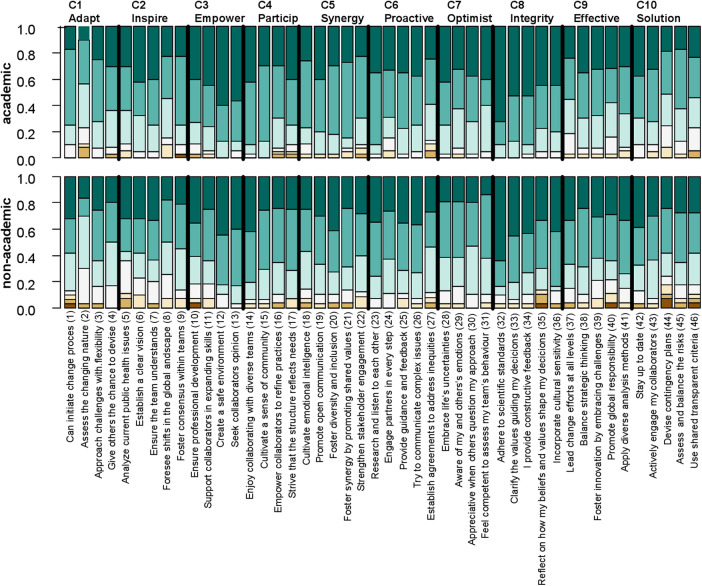
Perceived relevance of competencies by organisation. Distribution of ratings of perceived relevance of 46 leadership behaviours by organisation (academic vs. non-Academic). Each bar represents one leadership behaviour (see Box S3) rated on a 7-point Likert scale. Bar segments show the proportion of responses at each rating level (yellow = 1 not important, green = 7 very important).

Respondents ranked all ten transformational leadership competencies as relevant in their workplace; mean scores ranged from 5.58 to 6.23 on a 7-point Likert scale (Figure 1). Variation was minimal within each competency; internal consistency was high across all domains (Cronbach's alpha >0.77). Respondents rated Integrity (mean 6.23), Empowerment (mean 6.10), and Participation (mean 5.99) as most relevant in their current workplace. Perceived workplace relevance of competencies varied little across country income groups (mean scores from 5.24 to 6.4). When we compared workplace relevance of competencies in academic vs. non-academic sectors (Cohen's *d* range 0.05–0.47) (Figure 2) [see Supplementary Figure S6], government and NGOS vs. others (Cohen's *d* range 0.07 to 0.52) [see Supplementary Figure S7], and health care vs. non health care sectors (Cohen's *d* range 0.00–0.41) [see Supplementary Figure S8], we found small to moderate effect sizes. All competencies were rated as slightly more relevant in the workplace by respondents enrolled in PhD programs than by those only affiliated with a program (means ranged from 5.21 to 6.27); effect sizes were small to moderate [see Supplementary Figure S9]. Respondents in a PhD program rated Inspiration higher than those who did not participate in a PhD program (Cohen's *d* = 0.62, 95% CI 0.03–1.22), with moderate effect size. Mobility was associated with small to moderate differences in the perceived workplace relevance of competencies [see Supplementary Figure S13]. Respondents who remained in the same country reported slightly to moderately higher levels of workplace relevance across all competency dimensions than those who had moved countries at least once—before, during, or after the PhD, or due to their current main workplace. We observed the largest effect for Solution (Cohen's d = 0.44, 95% CI −0.04–0.91) but the CI was broad and included unity. Perceived importance of competencies differed only slightly by gender [see Supplementary Figure S11]. Men rated Adapt, Solution, Inspire, Proactive, Effective, and Optimist as slightly more relevant than women did; women rated Empower, Synergy, Integrity, and Participation higher. However, these observed gender differences were not statistically significant with the largest effect observed for Synergy (Cohen's d = 0.32, 95% CI −0.15–0.79).

Y axis: Proportion of Likert scale ratings

X axis: Competency descriptors

Y axis: Proportion of Likert scale ratings

X axis: Competency descriptors.

### Transformational leadership competencies developed during the PhD

Respondents rated all ten transformational leadership competencies as developed during their PhD training as a whole; mean ratings ranged from 5.29 to 5.97 on a 7-point Likert scale (Figure 1). Across competencies, score variability was low, and internal consistency was strong (Cronbach's alpha >0.77). Respondents rated these competencies as the ones they developed most intensively during PhD training: Integrity (mean 5.97); Participation (mean 5.69); and Empower (mean 5.61). The reported level of developed competencies differed across respondents' countries of employment depending on the country's income group; mean scores were between 4.56 and 6.21. All income groups rated Integrity as highest. Respondents in PhD programs reported to have significantly higher developed the competencies Adapt (Cohen's *d* = 0.74, 95% CI 0.14–1.34) and Optimist (Cohen's *d* = 0.60, 95% CI 0.00–1.19), with medium to large effect sizes, than graduates who had not completed a PhD program [see Supplementary Figure S10]. Mobility was associated with substantial differences in developed competencies [see Supplementary Figure S14]. Respondents who resided in the same place before, during and after the PhD and did not change their workplace, reported to have developed competencies significantly higher across all ten dimensions than those who had changed locations (Supplementary Figures S2, S4). For example, mean ratings for Solution were 5.85 vs. 4.53 (Cohen's *d* = 0.99, 95% CI 0.5–1.5) and, for Adapt, were 5.78 vs. 4.74 (Cohen's *d* = 0.89, 95% CI 0.39–1.38). We observed medium to large effects for other competencies: Effective (Cohen's *d* = 0.86, 95% CI 0.36–1.36); Synergy (Cohen's *d* = 0.81, 95% CI 0.32–1.3); Inspire (Cohen's *d* = 0.78, 95% CI 0.29–1.27); Proactive (Cohen's *d* = 0.77, 95% CI 0.27–1.26); Participate (Cohen's *d* = 0.76, 95% CI 0.27–1.25); Empower (Cohen's *d* = 0.68, 95% CI 0.2–1.17); Optimist (Cohen's *d* = 0.6, 95% CI 0.12–1.08); and, Integrity (*d* = 0.55, 95% CI 0.1–1.03). Women and men reported similar levels of competency development across all competencies.

### Alignment of workplace-relevant and PhD-developed transformational leadership competencies

**Figure 3:**
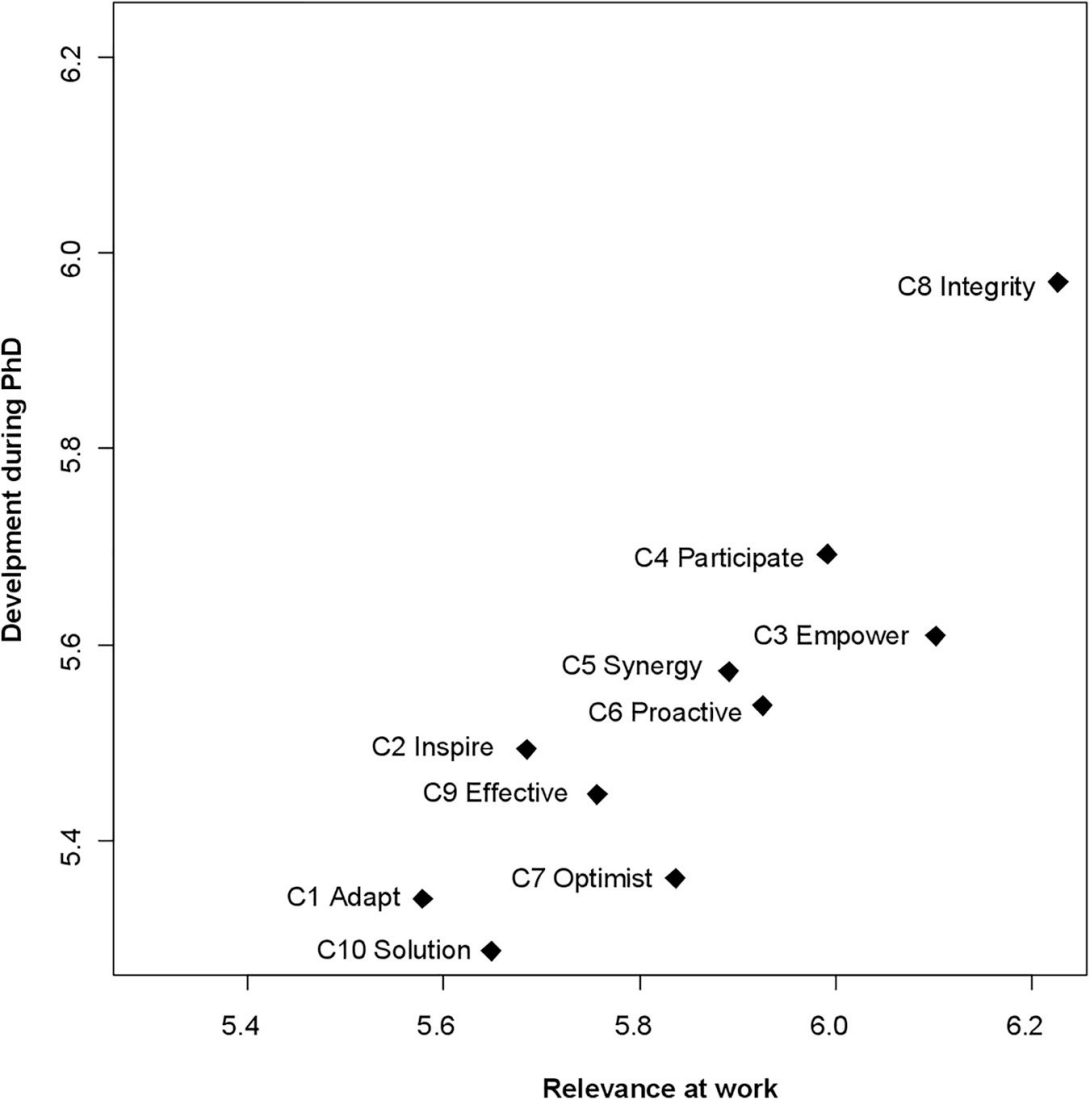
Competency relevance in the workplace versus development during PhD education. Each dot represents one competency positioned according to its mean Likert score (1–7) for perceived relevance in the workplace (x-axis) and perceived development during the PhD (y-axis).

For all ten transformational leadership competencies, respondents overall reported that the level of competencies relevant in the workplace exceeded those developed during PhD training as a whole (Figure 3). Effect sizes reached moderate levels for Empowerment (Cohen's *d* = 0.44, 95% CI 0.11–0.78), Optimist (Cohen's *d* = 0.41, 95% CI 0.08–0.75), Proactive (Cohen's *d* = 0.39, 95% CI 0.06–0.73) and Synergy (Cohen's *d* = 0.31, 95% CI −0.02–0.65), while the remaining six competencies showed small effects (Cohen's *d* ranging from 0.17 to 0.29) [see Supplementary Figure S5]. Exploratory analyses by the income level of respondents' countries of employment provided some evidence for differences between relevant and developed competencies. In low- and middle-income countries, differences between relevant and developed competencies were minor (Cohen's *d* = 0.02–0.29), but in high-income countries differences were moderate to large across competencies (Cohen's *d* = 0.30–0.77), with the largest differences for Proactive (Cohen's *d* = 0.77, 95% CI 0.23–1.32) and Empower (Cohen's *d* = 0.74, 95% CI 0.20–1.27). There were no relevant gender differences for relevant vs. developed competencies (Cohen's *d* < 0.32); gender did not meaningfully influence self-perceived relevant and developed competencies in our sample [see Supplementary Figures S11, S12].

## Discussion

Across professional working areas and country income groups, respondents rated all ten transformational leadership competencies as relevant, with similar patterns across settings. PhD program graduates rated all competencies as slightly more relevant in the workplace than graduates unaffiliated with a PhD program. Graduates reported that their PhD training helped them develop all competencies. Those from low- and middle-income countries reported higher levels of developed competencies during their PhD training. They also reported more alignment between relevant competencies in their workplace and developed competencies during their PhD training than graduates from high-income countries. Mobility was associated with small to moderate differences in perceived relevance of competencies in the workplace and substantial differences in the reported level of developed competencies. Participants who remained in the same location throughout the PhD reported significantly higher levels of competency development across all ten dimensions than those who moved. There was no evidence that gender was associated with relevance or development of competencies. The relatively low response rate means that these findings should be considered exploratory and interpreted with appropriate caution.

Our findings indicate that workplace requirements for transformational leadership are consistent across country income groups. This aligns with Frenk et al.'s view (9) that while local contexts shape how competencies are enacted, certain core professional capabilities remain universal. In particular, the ability to manage complex health systems and respond to global health challenges is essential across all settings. Our findings align with previous findings that emphasized leadership-related competency development within doctoral training programs (1, 8–10, 12, 13, 17). We observed higher self-reported competency development among graduates in low- and middle-income countries, which challenges assumptions that doctoral preparation is uniform across global contexts (1, 2). Yet, this pattern aligns with the findings of Crede et al.'s meta-analysis (22), which highlights the context-dependent nature of transformational leadership behaviors. Specifically, the analysis showed that such behaviors tend to have stronger effects in lower-income settings than in higher-income contexts, including Western Europe and North America. Previous studies suggested that international and temporary mobility can improve leadership competency development by expanding professional networks, fostering intercultural awareness, and creating opportunities for collaboration in new environments (23–25). In contrast to these studies, participants in our study who remained in the same place throughout their PhD reported higher levels of competency development across all ten leadership dimensions. This finding should be interpreted cautiously, as the cross-sectional design and self-reported measures do not allow causal conclusions. Alternative explanations may include differences in supervision, institutional support, program structures, or personal circumstances. Our gender-related findings align with research that found no significant differences in self-perceived transformational leadership competencies (26). Other research does suggest that gendered patterns may emerge over the course of a career: women's self-perceived transformational leadership declined as they advanced, while men's increased, perhaps as a result of facing structural and contextual barriers (27, 28). The widening gap in the self-perception is also mirrored in the persistent gender disparities in leadership in academic public health (29). Our results align with Trechsel's (30) findings, where structured PhD programs improved competency development compared to more open doctoral formats. Programs like CARTA, which operate in low- and middle-income settings and are rooted in multidisciplinary and experiential pedagogies, may help participants develop stronger transformational leadership competencies (31–33).

We recommend adopting a transformational leadership lens as a guiding framework for doctoral education in global health and workforce development to strengthen the ability to balance competing demands and respond adaptively to changing contexts (15). According to Abimbola (3) global health often involves supporting “someone else”, creating an inherent power imbalance and information gap: those designing interventions may lack context-specific knowledge, while target populations may have limited ability to influence decisions. Transformational leadership development may help bridge these gaps through a shift from isolated courses to more integrated developmental pathways embedded in curricula, pedagogy, and collaborative practices (12, 30, 34–36). For curriculum design, leadership development could be integrated throughout the PhD journey rather than being offered as optional or stand-alone training. This could be achieved through experiential learning, interdisciplinary teamwork, reflective practice, mentoring and coaching, peer learning, and engagement with real-world leadership challenges. For example, reflective journals and coaching sessions can strengthen self-awareness and self-regulation, thereby fostering self-leadership*.* Embedding leadership competencies within research activities and collaborative projects may help doctoral candidates connect leadership development to their academic and professional practice. For example, students could lead an interdisciplinary team project with structured reflection and feedback integrated into the course assessment. Funders could strengthen leadership development by recognizing it as a core research competency and incorporating a leadership development plan into PhD and postdoctoral grant applications. Applicants could be asked to outline how they will develop leadership competencies during the funding period through mentoring, team leadership, interdisciplinary collaboration, and other professional development activities. This could then encourage interconnected, complementary partnerships that foster leadership and mutual learning across diverse settings (37). Stronger alignment with labor market needs could be achieved through co-created programs involving academia and professional sectors, complemented by early-career leadership onboarding from employers. Sustained mentoring, and collaboration between academia and practice reinforce key competencies such as Integrity, Empowerment, and Adaptability, translating frameworks into effective leadership in practice (1, 9, 13, 38–40). At the program level, strengthening educator and supervisor capacity through training in adult learning and transformative pedagogies supports locally relevant knowledge production and sustainable institutional development. This could include training faculty to replace traditional lectures with facilitated workshops in which PhD students collaboratively solve authentic research leadership challenges. Fostering respectful and supportive learning environments within PhD programs can strengthen leadership development while reinforcing values of human dignity, mutual respect, and professional integrity. For example, supervisory relationships characterized by constructive feedback, psychological safety, and mutual respect can help doctoral candidates develop confidence, autonomy, and leadership competencies (14)*.* At the individual level, leadership development might be approached through scaffolded learning pathways that combine experiential learning, guided reflection, mentoring or coaching, and peer learning (33, 35, 41–44). For instance, students coordinating multicentre data collection could receive structured mentoring before and after the collaboration to reflect on communication, delegation, and team coordination. Providing safe, reflective spaces, targeted support for mobile PhD candidates, and explicit integration of leadership into research practice enhances competency development (12, 30, 34). One practical approach is to establish confidential monthly reflection groups in which PhD students can openly discuss real leadership challenges encountered during their doctoral training, reflect on their experiences, learn from peers, and receive constructive feedback in a psychologically safe environment without formal assessment.

As recently emphasized by Behbob and Czabanowska (45) research is still needed to explore and strengthen approaches to leadership capacities. Future research should include larger and more geographically diverse samples. Studies should capture variation across multiple countries within regions. Greater inclusion from underrepresented regions is needed to assess representativeness and improve generalizability. We recommend adopting comparative and longitudinal designs to disentangle program-specific effects and account for responses at different stages of participants' careers. Mixed-methods approaches may make it possible to better interpret competencies across cultural and multidisciplinary contexts. We also suggest exploring how curricula can integrate expertise from low-income settings and how reciprocal partnerships can support practice-informed leadership development. Further work could examine how transformational leadership might be embedded in doctoral training (30) and reinforced through postgraduate or workplace-based learning. Cross-sector comparisons may help identify both transferable and context-specific competencies. In addition, the extent to which undergraduate leadership programs can complement and strengthen leadership development at the PhD level could also be investigated (46).

### Limitations

Our study had several limitations. Generalizability is limited by our small sample size, and our focus on five PhD programs with heterogeneous structures. Because we did not include programs from North and South America or Asia, our findings apply mainly to European and African graduates. Internal validity may be compromised by differences in time since graduation, since timing can influence recall and career-related responses. The low response rate (estimated 11%) may have introduced non-response, selection, and survivorship bias, as individuals with a stronger interest in leadership development and more positive program experiences may have been more likely to participate. This may have influenced the findings and limits the generalizability of the results. In addition, our findings rely entirely on self-reported perceptions of competency development and workplace relevance. Such measures are subject to recall bias, particularly because participants completed their PhDs a median of six years before the survey and may not have accurately remembered the extent to which specific competencies were developed during their training. Self-reports may also have been influenced by social desirability bias, potentially leading participants to overestimate competency development or the relevance of competencies in their professional roles. Furthermore, we could not ensure that survey items were interpreted consistently across cultural and disciplinary contexts. Finally, the same participant may have filled out duplicate survey entries, though we used a single survey link to reduce the chance of duplication.

## Conclusion

Our findings indicate that, while transformational leadership competencies are widely valued in global health, graduates do not consistently perceive that these competencies were developed during their PhD training, particularly in high-income countries. Reported competency development varied across geographic and organizational settings, which may reflect differences in educational structures, institutional priorities, and local contexts. Programs with formally structured leadership-related educational components and dedicated pedagogical approaches were associated with higher levels of self-reported competency development. However, the cross-sectional nature of the study does not permit conclusions about causality or effectiveness. These findings suggest that transformational leadership may be a useful framework for informing doctoral education in global health and workforce development more broadly. They also highlight the potential value of integrating leadership development throughout the entire PhD journey. Future research should explore how different educational approaches contribute to leadership competency development and how lessons from diverse contexts, including low-income settings, can inform efforts to strengthen PhD training and transformational leadership development in global health.

## Data Availability

The raw data supporting the conclusions of this article will be made available by the authors, without undue reservation.
