# Supplementary material for: An exploratory study on transformational leadership competencies in global health
Source: Front Health Serv. 2026 Aug 4;6:1876328. doi: 10.3389/frhs.2026.1876328 (PMC13481433; doi:10.3389/frhs.2026.1876328)
Supplement: Supplementary file 1 [file Table1.docx]

**Supplementary material**

Table of Contents

[Figure S1: Survey Response Flow Chart	2](#_Toc227240189)

[Box S1: PhD programs from which we recruited graduates to the survey	3](#_Toc227240190)

[Box S2: Executive summary: Delphi study on transformational leadership in public health	4](#_Toc227240191)

[Box S3: Final transformational leadership competencies	5](#_Toc227240192)

[Table S1 Leadership training	6](#_Toc227240193)

[Figure S2 Mobility across country income groups during PhD trajectory	6](#_Toc227240194)

[Figure S3: Matrix of thematic overlaps between participants’ main work areas	7](#_Toc227240195)

[Figure S4: Place of residence before, during, and after the PhD, and current work location	8](#_Toc227240196)

[Table S2: Universities and PhD Programs	9](#_Toc227240197)

[Box S4: Country classification (nationality)	10](#_Toc227240198)

[Figure S5: Differences between developed and relevant leadership competencies (Cohen’s d with 95% confidence intervals)	11](#_Toc227240199)

[Figure S6: Differences in leadership competencies between academic and non-academic employment (Cohen’s d with 95% confidence intervals)	12](#_Toc227240200)

[Figure S7: Differences in transformational leadership competencies between NGO and other employment sectors (Cohen’s d with 95% confidence intervals)	13](#_Toc227240201)

[Figure S8: Differences in leadership competencies between health care and other employment sectors (Cohen’s d with 95% confidence intervals)	14](#_Toc227240202)

[Figure S9: Comparison of relevant transformational leadership competencies in PhD programs versus no formal program (Cohen’s d, 95% CI)	15](#_Toc227240203)

[Figure S10: Comparison of developed transformational leadership competencies in PhD programs versus no formal program (Cohen’s d, 95% CI).	16](#_Toc227240204)

[Figure S11: Gender differences in relevant transformational leadership competencies (Cohen’s d, 95% CI)	17](#_Toc227240205)

[Figure S12: Gender differences in developed transformational leadership competencies (Cohen’s d, 95% CI)	18](#_Toc227240206)

[Figure S13: Relevant competencies by mobility status (mobile vs. non-mobile, Cohen’s d, 95% CI)	19](#_Toc227240207)

[Figure S14: Developed competencies by mobility status (mobile vs. non-mobile, Cohen’s d, 95% CI)	20](#_Toc227240208)

# Figure S1: Survey Response Flow Chart

# Box S1: PhD programs from which we recruited graduates to the survey

- **Consortium for Advanced Research Training in Africa (CARTA)**

CARTA is a structured, pan‑African doctoral training program that strengthens research capacity and leadership in public and population health. CARTA provides PhD fellowships for early-career faculty at African universities, including four interdisciplinary Joint Advanced Seminars (JAS), mentorship, professional-skills training, and networking opportunities. The program emphasises multidisciplinary approaches to health challenges and supports fellows throughout the full doctoral training period (see [cartafrica.org](https://cartafrica.org/?utm_source=chatgpt.com)).

- **Care and Public Health Research Institute (CAPHRI), Maastricht University**

CAPHRI hosts a structured PhD Graduate School where doctoral candidates conduct individual research projects with high-quality supervision. CAPHRI emphasises transdisciplinary research and societal relevance and offers methodological and transferable‑skills training (see [maastrichtuniversity.nl](https://www.maastrichtuniversity.nl/research/school-caphri-care-and-public-health-research-institute/phd-graduate-school-caphri?utm_source=chatgpt.com)).

**PhD Program Health Sciences (PPHS), University of Basel / Swiss TPH**

The PPHS is a structured doctoral program at the University of Basel (in collaboration with Swiss TPH), open to PhD students in epidemiology, public health, clinical research, nursing science, drug development, and related fields. PPHS provides courses, networking, career support, and a PPHS certificate (see [unibas.ch/pphs](https://pphs.unibas.ch/?utm_source=chatgpt.com)).

**Swiss School of Public Health PhD Program in Public Health (PPH)**

Managed by the Swiss School of Public Health (SSPH+), this inter-university PhD program offers structured training in public health across partner Swiss universities. It integrates research, coursework, mentorship, and prepares candidates for both academic and applied public health leadership (see [ssphplus.ch](https://ssphplus.ch/en/research/phd-programs/?utm_source=chatgpt.com)).

- **Swiss School of Public Health Inter‑university Graduate Campus (IGC)**

The SSPH+ IGC are a central hub for doctoral training: methods courses, topical public health training, soft‑skills workshops, networking, and career development. Open to PhD and MD students supervised by SSPH+ faculty, it also offers a certificate in public health (see [ssphplus.ch](https://ssphplus.ch/en/graduate-campus/?utm_source=chatgpt.com)).

# Box S2: Executive summary: Delphi study on transformational leadership in public health

The Delphi study aimed to develop a consensus-based competency framework for transformational leadership in public health. Recognizing the complex and dynamic nature of public health systems, the study sought to define core competencies along with behavioural descriptors that could guide leadership development, assessment, and practical application across diverse organizational and cultural contexts. The study employed a five-round modified Delphi design, combining three asynchronous online surveys with two synchronous online workshops. An international panel of approximately 60 experts from around 30 countries participated, representing sectors such as education, research, policy, human resources, and healthcare organizations. The process began with a literature review, which identified 38 preliminary competencies. These competencies were iteratively refined by the expert panel through rating, ranking, and development of specific behavioural descriptors, while the workshops provided opportunities for interactive discussion and consensus building.

The final framework consists of 10 core transformational leadership competencies:

1. Adapts to the needs of the ecosystem
2. Inspires others to commit to common goals
3. Empowers others to capture and build upon their competencies and wisdom
4. Practices participatory and inclusive leadership
5. Creates synergies and fosters a collaborative environment
6. Proactively manages partnerships with diverse actors
7. Personifies optimism to build resilience and perseverance in challenging times
8. Acts with integrity
9. Operates effectively within and across complex and multifaceted systems
10. Pursues strategic approaches for value-based, innovative solutions

To facilitate practical application, the authors developed a four-stage developmental model, conceptualizing leadership progression from competent to transformational. Individuals assess the frequency with which they demonstrate the behaviors described. The framework provides both a guide for self-development and a structure for organizational training and evaluation.

For educators and curriculum designers, the framework offers a structured yet adaptable tool to design leadership training programs that are sensitive to local and organizational contexts. For public health organizations, it provides a means to identify competency gaps, guide professional development, inform recruitment and performance assessment, and enhance overall workforce effectiveness. By integrating context-sensitive competencies with practical behavioural descriptors, this framework contributes to bridging the gap between leadership education and real-world public health practice, while fostering transformational leadership capable of navigating complex, evolving health systems.

From: Bürkin BM, Czabanowska K, Babich M., et al. Competencies for transformational leadership in public health—An international Delphi consensus study. International Journal of Public Health 2024; 69:1606267.doi: <https://doi.org/10.3389/ijph.2024.1606267>

# Box S3: Final transformational leadership competencies

| **C1 Adapts according to the needs of the eco-system** | **C6 Proactively manages partnerships with diverse actors** |
| --- | --- |
| 1. I can initiate change processes in a competent manner and guided by principles 2. I assess the changing nature of global health, the main factors that influence it, and the availability of resources. 3. I approach challenges with flexibility, a strong understanding of processes, and alignment between new information, observations, and past experiences. 4. I give others the chance to devise new approaches and take the initiative. | 23. I research and listen to each partner’s interests. 24. I engage partners in every step of the process, first in informal settings to build trust and camaraderie, and then in formal settings. 25. I provide guidance and feedback on collaborators’ ideas, concerns, or challenges. 26. I try to communicate complex issues clearly and credibly to widely varied audiences. 27. I establish agreements that advance cross-sectoral, international initiatives to address inequities, implement interventions, and ensure transparency in partnerships. |
| **C2 Inspires others to commit to common goals** | **C7 Personifies optimism to build resilience and perseverance in challenging times** |
| 5. I analyse current public health issues and their determinants, considering political, environmental, and economic dynamics to ensure a comprehensive understanding. 6. I establish a clear vision of project goals and desired outcomes before initiating any work. 7. I ensure that the team understands our goals as a collective. 8. I foresee shifts in the global landscape, identifying changes in partnerships and sectors, and assess their impact on our goals to ensure adaptability. 9. I foster consensus within teams and external partners, aligning shared aspirations and benefits. | 28. I embrace life’s uncertainties, viewing challenges as opportunities for learning and growth with a solution-oriented mindset. 29. I am aware of my and others’ emotions, and I cope with negative emotions and impulses. 30. I am appreciative when others question or challenge my approach remaining objective in negotiations. 31. I feel competent to assess my team’s behaviour in different contexts and situations. |
| **C3 Empowers others to fully capture and build upon their competencies and wisdom** | **C8 Acts with integrity** |
| 10. I ensure professional development opportunities are available to members of my organization (e.g., training, mentoring, peer advising, coaching). 11. I support collaborators in expanding their professional skills according to their individual strengths. 12. I create a safe environment that fosters a respectful work culture. 13. I seek collaborators’ opinions and ideas to enhance teamwork. | 32. I adhere to scientific standards and respect research integrity. 33. I clarify the values guiding my decisions and actions. 34. I provide constructive feedback tailored to each individual. 35. I reflect on how my beliefs and values shape my decisions. 36. I incorporate cultural sensitivity and ethical principles in my interactions. |
| **C4 Practices participatory and inclusive leadership** | **C9 Operates effectively within and across complex and multifaceted systems** |
| 14. I enjoy collaborating with diverse teams to co-create projects from inception to evaluation. 15. I cultivate a sense of community by recognizing shared values, celebrating achievements, and bridging diverse perspectives. 16. I empower collaborators to refine our practices while fostering accountability through shared decision-making. 17. I strive to ensure that the structure of my team and our activities reflect the composition and needs of the communities we aim to serve. | 37. I lead change efforts at all levels, inspiring others to embrace innovation and recognizing the benefits of approaching challenges differently. 38. I balance strategic thinking with attention to detail, breaking down large problems into manageable tasks to address both patterns and specifics. 39. I foster innovation by embracing challenges, refreshing perspectives, and identifying opportunities for systemic solutions. 40. I promote global responsibility by linking local actions to global impacts and encouraging collaborators to integrate these perspectives in decision-making. 41. I apply diverse analysis methods to draw conclusions, encouraging others to integrate seemingly unrelated ideas to develop holistic solutions. |
| **C5 Creates synergies and fosters a collaborative environment** | **C10 Pursues strategic approaches for value-based, innovative solutions”** |
| 18. I cultivate emotional intelligence by valuing individual perspectives and cultural norms. 19. I promote open communication to support the resolution of conflicts. 20. I foster diversity and inclusion by valuing individuality, building common ground, and creating culturally sensitive teams. 21. I foster synergy by promoting shared values, integrating diverse strengths, and leading collaborative initiatives. 22. I strengthen stakeholder engagement in addressing global issues through interdisciplinary workshops and knowledge exchange. | 42. I stay up to date with and remain sensitive to changing external trends. 43. I actively engage my collaborators or colleagues in discussions and adherence to agreed-upon   values and standards.   44. I devise contingency plans for potential global challenges. 45. I assess and balance the risks, benefits, and unforeseen consequences of our work. 46. I use shared, transparent criteria to direct strategic organizational decision-making and planning. |

# Table S1 Leadership training

| **Leadership training (LT) during and after PhD** | **n** | **Percentages of respondents**  **(N=71)** |
| --- | --- | --- |
| **LT during PhD** |  |  |
| - Yes | **29** | 40.8% |
| - No | 42 | 59.2% |
|  |  | **(N=29)** |
| **Location of LT during PhD** |  |  |
| - University/PhD program | 21 | 72.4% |
| - Elsewhere* | 10 | 34.5% |
| **Type of LT during PhD** | | |
| - Courses/Seminars | 21 | 72.4% |
| - Lectures | 11 | 37.9% |
| - Coaching | 4 | 13.8% |
| - Other | 3 | 10.3% |
| **Leadership training (LT) after PhD** | | |
| - Yes | **25** | 35.2% |
| - No | 46 | 64.8% |
| **Motivation for LT** | | |
| - Offered by employer | 15 | 60% |
| - Interest in subject area | 12 | 48% |
| - Other* | 2 | 8% |
| **Type of LT after PhD** | | |
| - Courses/Seminars | 18 | 72% |
| - Lectures | 5 | 20% |
| - Coaching | 7 | 28% |
| - Other | 2 | 8% |

# Figure S2 Mobility across country income groups during PhD trajectory

Y-axis: Percentage of graduates

X-axis: Career stage (before PhD | during PhD | after PhD | current

Participants’ mobility across income group categories (low-, lower-middle-, upper-middle- and high-income countries) over four career stages (before, during, and after the PhD, and in their current position). Flow width indicates the proportion of participants who transitioned between income groups

# Figure S3: Matrix of thematic overlaps between participants’ main work areas

# Figure S4: Place of residence before, during, and after the PhD, and current work location

| Residence before PhD | Residence after PhD |
| --- | --- |
|  |  |
| Residence during PhD | **Residence of current work** |
|  |  |

# Table S2: Universities and PhD Programs

**University**

**Africa**

1. Kenya: Moi University; University of Nairobi
2. Malawi: University of Malawi
3. Nigeria: Obafemi Awolowo University; University of Ibadan
4. Ruanda: University of Ruanda
5. South Africa: University of the Witwatersrand
6. Uganda: Makerere University

**Europe**

7. Switzerland: Bern University of Applied Sciences (BFH); Eidgenössische Technische Hochschule Zürich (ETHZ); École Polytechnique Fédérale de Lausanne (EPFL) ; Haute école spécialisée de Suisse occidentale (HES-SO) ; Haute école de santé de Genève (HEdS) ; University of Applied Sciences and Arts of Southern Switzerland (SUPSI); University of Basel; University of Bern; University of Fribourg; University of Geneva; University of Lausanne; University of Lucerne; University of Neuchâtel; Università della Svizzera italiana; University of Zurich; Zurich University of Applied Sciences (ZHAW)
8. The Netherlands: Maastricht University

**PhD Programs**

1. Consortium for Advanced Research Training in Africa (CARTA)
2. Care and Public Health Research Institute (CAPHRI), Faculty of Health, Medicine & Life Sciences, Maastricht University
3. PhD Program Health Sciences (PPHS), University of Basel, Swiss TPH
4. Swiss School of Public Health PhD Program in Public Health (PPH)
5. Swiss School of Public Health Inter-university Graduate Campus (IGC) with certificate (former)
6. Other, please specify
7. No structured program

# Box S4: Country classification (nationality)

| **Nationality** |  |
| --- | --- |
| Rwanda | 4 |
| Kenya | 10 |
| India | 2 |
| USA | 2 |
| Nigeria | 7 |
| Switzerland | 14 |
| South Africa | 1 |
| Ghana | 1 |
| Tanzania | 5 |
| Burkina Faso | 2 |
| Uganda | 3 |
| Zimbabwe | 1 |
| Mongolia | 2 |
| Vietnam | 1 |
| Lebanon | 1 |
| France | 1 |
| Swaziland (Eswatini) | 1 |
| China | 1 |
| Malawi | 3 |
| Albania | 1 |
| Sudan | 1 |
| Denmark | 1 |
| Italy | 1 |
| Ethiopia (currently in a temporary status of unclassification for FY26) | 2 |
| Bangladesh | 1 |
| Mali | 1 |
| Cameroon | 1 |
| **Total** | **71** |

| **Classification countries** | |
| --- | --- |
| **LOW-INCOME ECONOMIES ($1,135 OR LESS)** | |
| Rwanda  Burkina Faso  Malawi  Uganda  Sudan  Mali | 16  (including Ethiopia) |
| **LOWER-MIDDLE INCOME ECONOMIES ($1,136 TO $4,495)** | |
| Kenya  India  Nigeria  Ghana  Tanzania  Zimbabwe  Vietnam  Lebanon  Swaziland  Bangladesh  Cameroon | 31 |
| **UPPER-MIDDLE-INCOME ECONOMIES ($4,496 TO $13,935)** | |
| South Africa  Mongolia  China  Albania | 5 |
| **HIGH-INCOME ECONOMIES ($13,935 OR MORE)** | |
| USA  Switzerland  France  Denmark  Italy | 19 |
| **Total** | **71** |

# Figure S5: Differences between developed and relevant leadership competencies (Cohen’s d with 95% confidence intervals)

Legend: Standardized mean differences (Cohen’s d) between developed and relevant transformational leadership competencies; points indicate effect sizes; horizontal lines represent 95% confidence intervals (CI); the vertical line at zero indicates no difference.

# Figure S6: Differences in leadership competencies between academic and non-academic employment (Cohen’s d with 95% confidence intervals)

Legend: Standardized mean differences (Cohen’s d) in transformational leadership competencies between academic and non-academic employment; points indicate effect sizes; horizontal lines represent 95% confidence intervals (CI); the vertical line at zero indicates no difference.

# Figure S7: Differences in transformational leadership competencies between NGO and other employment sectors (Cohen’s d with 95% confidence intervals)

Legend: Standardized mean differences (Cohen’s d) in transformational leadership competencies between NGO and other employment sectors; points indicate effect sizes; horizontal lines represent 95% confidence intervals (CI); the vertical line at zero indicates no difference.

# Figure S8: Differences in leadership competencies between health care and other employment sectors (Cohen’s d with 95% confidence intervals)

Legend: Standardized mean differences (Cohen’s d) in transformational leadership competencies between health care and other employment sectors; points indicate effect sizes; horizontal lines represent 95% confidence intervals (CI); the vertical line at zero indicates no difference.

# Figure S9: Comparison of relevant transformational leadership competencies in PhD programs versus no formal program (Cohen’s d, 95% CI)

Legend: Standardized mean differences (Cohen’s d) in relevant transformational leadership competencies between PhD program participation versus no formal program; points indicate effect sizes; horizontal lines represent 95% confidence intervals (CI); the vertical line at zero indicates no difference.

# Figure S10: Comparison of developed transformational leadership competencies in PhD programs versus no formal program (Cohen’s d, 95% CI).

Legend: Standardized mean differences (Cohen’s d) in developed transformational leadership competencies between PhD program participation versus no formal program; points indicate effect sizes; horizontal lines represent 95% confidence intervals (CI); the vertical line at zero indicates no difference.

# Figure S11: Gender differences in relevant transformational leadership competencies (Cohen’s d, 95% CI)

Legend: Standardized mean differences (Cohen’s d) in relevant transformational leadership competencies between female versus male; points indicate effect sizes; horizontal lines represent 95% confidence intervals (CI); the vertical line at zero indicates no difference.

# Figure S12: Gender differences in developed transformational leadership competencies (Cohen’s d, 95% CI)

Legend: Standardized mean differences (Cohen’s d) in developed transformational leadership competencies between female versus male; points indicate effect sizes; horizontal lines represent 95% confidence intervals (CI); the vertical line at zero indicates no difference.

# Figure S13: Relevant competencies by mobility status (mobile vs. non-mobile, Cohen’s d, 95% CI)

Legend: Standardized mean differences (Cohen’s d) in relevant transformational leadership competencies between mobile and non-mobile graduates; points indicate effect sizes; horizontal lines represent 95% confidence intervals (CI); the vertical line at zero indicates no difference. Mobility status: Non-mobile graduates remained in the same location before, during, and after the PhD and in their current main workplace. Mobile graduates changed their place of residence or main workplace at least once before, during, or after the PhD.

# Figure S14: Developed competencies by mobility status (mobile vs. non-mobile, Cohen’s d, 95% CI)

Legend: Standardized mean differences (Cohen’s d) in developed transformational leadership competencies between mobile and non-mobile graduates; points indicate effect sizes; horizontal lines represent 95% confidence intervals (CI); the vertical line at zero indicates no difference. Mobility status: Non-mobile graduates remained in the same location before, during, and after the PhD and in their current main workplace. Mobile graduates changed their place of residence or main workplace at least once before, during, or after the PhD.
